# A case series describing the epidemiology and clinical characteristics of COVID-19 infection in Jilin Province

**DOI:** 10.1080/21505594.2020.1767357

**Published:** 2020-05-22

**Authors:** Na Du, Haiying Chen, Qing Zhang, Lihe Che, Lixin Lou, Xiaohua Li, Kaiyu Zhang, Wanguo Bao

**Affiliations:** Infectious Diseases Department, The First Hospital of Jilin University, Changchun, China

**Keywords:** COVID-19, severe acute respiratory syndrome, SARS-CoV-2, clinical characteristic, epidemiology, Jilin

## Abstract

Since its outbreak in Wuhan, Hubei Province China, 2019-coronavirus infected disease (COVID-19) had been widely spread all over the world, the control of which calls for a better understanding of its epidemiology and clinical characteristics. We included 12 confirmed cases of COVID-19 in First Affiliated Hospital of Jilin University from 23 January 2020 to 11 February 2020, which were retrospectively analyzed for epidemiological, demographic, clinical, laboratory, and radiological features. All the patients were confirmed by nucleic acid detection, the average age of whom was 45.25 years (range, 23–79 years). Most patients had a history of Wuhan traveling or had contact with Wuhan travelers or infected cases. Obvious family cluster was observed. Clinical manifestations included fever (12/12), fatigue (10/12), cough (6/12), sore throat (4/12), headache (3/12), and diarrhea (2/12). Only three out of eight patients had pneumonia manifestation on radiography. Most patients had a normal white blood cell (WBC) count and normal or reduced lymphocyte (LY) count. Pneumonia changes were observed in all the four patients who underwent a chest CT scan. Only one elderly patient developed severe pneumonia, while all the rest were mild disease and had a self-limiting course.

## Introduction

Since the outbreak of a novel coronavirus pneumonia in Wuhan, Hubei Province China in December 2019, the disease has rapidly spread to many cities within China and globally [1,2]. The cause is the novel coronavirus SARS-CoV-2 (severe acute respiratory syndrome coronavirus 2; formerly known as 2019-nCoV) [3], and it is capable of rapid human-to-human transmission. The World Health Organization (WHO) has termed the pneumonia caused by SARS-CoV-2 as 2019-coronavirus infected disease (COVID-19) [3,4]. COVID-19 is believed to be a zoonotic disease that originated from Huanan Seafood Wholesale Market, although the intermediate host remains a puzzle [5]. The main routes include air, respiratory droplet, contact, and even fecal-oral route [6].

The number of suspected or confirmed cases and mortalities due to COVID-19 has continued to rise in Wuhan as well as in many other areas [7]. By 16 February 2020, Jilin Province had reported 89 cases confirmed by nucleic acid detection, among which 12 were diagnosed at First Affiliated Hospital of Jilin University.

There are still few reports regarding COVID-19 outside Wuhan. This article describes the epidemiology and clinical features of 12 cases of COVID-19 treated at the First Affiliated Hospital of Jilin University in Changchun City, Jilin Province.

## Methods

From 23 January 2020 to 11 February 2020, we collected throat swabs from 109 outpatients suspected of SARS-CoV-2 infection and sent the swabs to the Disease Prevention and Control Center of Jilin Province for nucleic acid detection. Among these, 12 patients were reported as positive and were admitted to the Infection Department of First Affiliated Hospital of Jilin University. All the admitted patients underwent blood routine examination and lung computed tomography (CT); some underwent and chest radiography. Epidemiological, demographic, clinical, laboratory, radiological, and treatment data were collected.

The study was approved by the Ethics Committee of First Affiliated Hospital of Jilin University.

## Results

**Figure 1.**
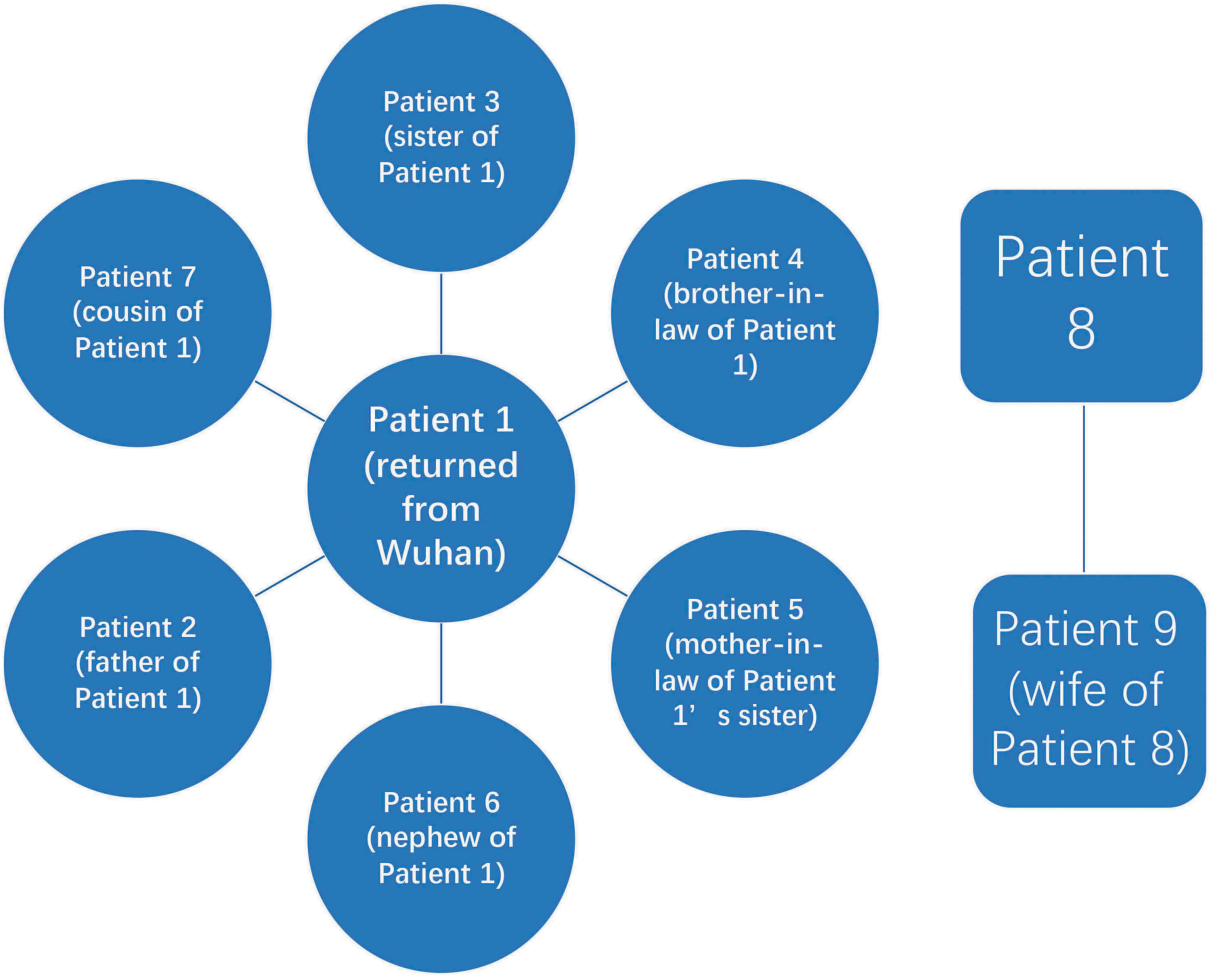
Epidemiological features of familial cluster.

The median age of the 12 hospitalized patients was 45.25 years (range, 23–79 years). Seven (54.3%) were men. Most of the patients had a history of traveling to Wuhan (patient [PT] 1 and PT 10) or to people who had traveled to Wuhan (PTs 2–7), or to patients with confirmed infections (PT 11, PT 12). The exposure history of two patients was unknown (PT 8 and PT 9). Epidemiological familial cluster was observed among these patients (Figure 1).

On admission, all 12 patients presented with fever (37.3–39.4°C) of 3 to 7 days. Other symptoms included fatigue, cough, sore throat, headache, and diarrhea in 10, 6, 4, 3, and 2 patients, respectively. Blood routine test was performed for all patients on admission. Most patients had a normal white blood cell (WBC) count and normal or reduced lymphocyte (LY) count (Table 1).

**Table 1.** Blood routine results of PTs 1 through 12.

	WBC	LY	HGB	RBC	PLT
1	5.67	1.43	127	3.97	235
2	4.32	0.46↓ *	135	4.34	197
3	6.87	1.20↓ *	143	4.03	187
4	7.43	1.67	156	4.56	156
5	4.17	1.29	121	4.53	183
6	4.23	1.34	156	4.32	128
7	3.98	1.22	137	3.89	207
8	6.46	1.56	129	3.87	267
9	7.45	1.35	126	4.68	189
10	5.76	1.32	158	3.59	166
11	4.09	1.15	148	3.77	154
12	4.36	1.54	139	3.65	148

* ↓ indicates lower than the normal range.

HGB, hemoglobin concentration; LY, lymphocyte; PLT, platelet count; RBC, red blood cell count; WBC, white blood cell count.

**Figure 2.**
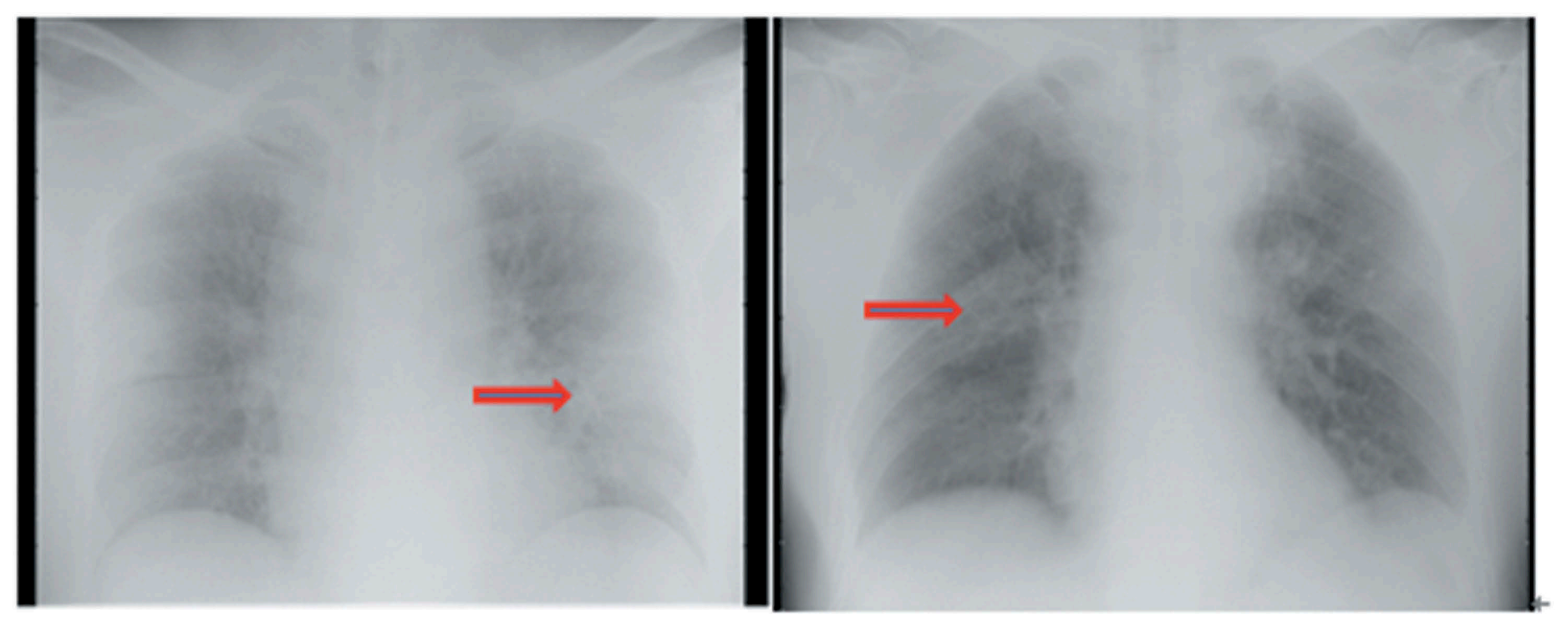
Typical change on chest radiographs of PT 10. The brightness of both lungs was decreased. Multiple patchy shadows with blurred edges were observed.

**Figure 3.**
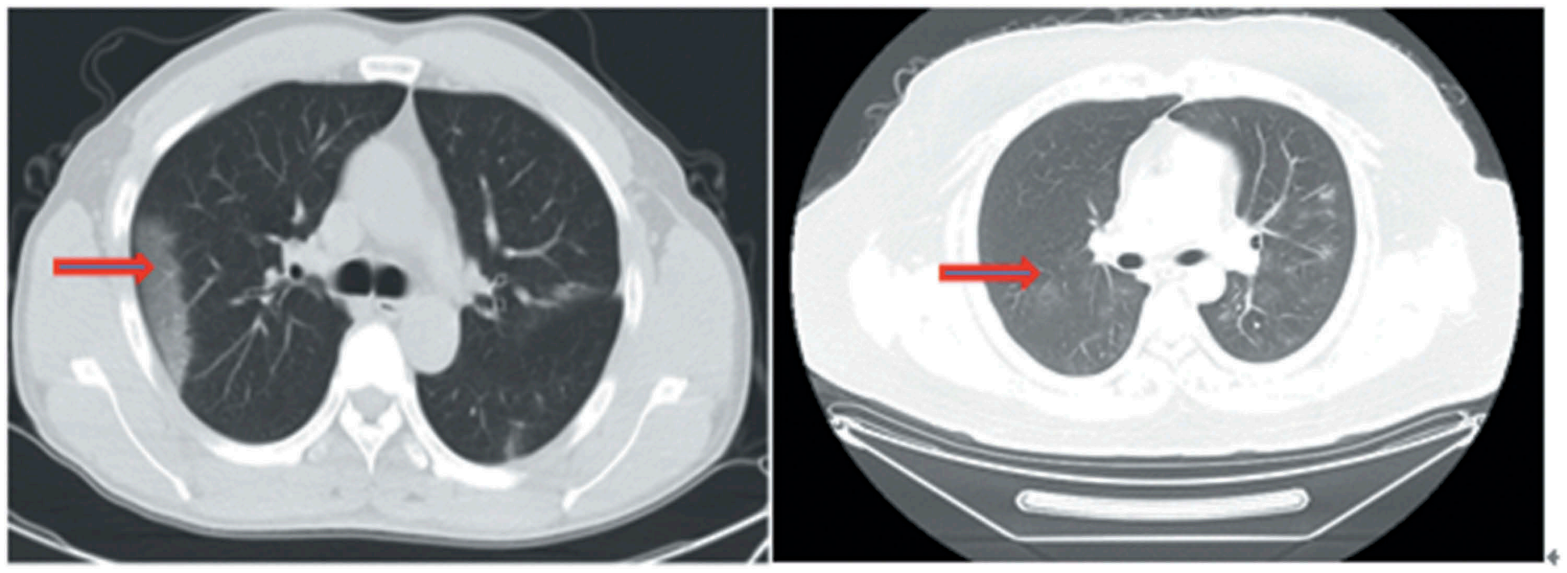
Typical changes in non-contrast enhanced chest CT scan. Multiple peripheral patchy ground-glass opacities with obscure boundary were seen in bilateral multiple lobes. Condensation shadow was observed on the lower right lobe.

On admission, chest x-ray was performed for eight patients. Three patients had typical viral pneumonia manifestations, such as multiple mottling and decrease of brightness (Figure 2), while no obvious abnormal change was observed on the radiography of the remaining five patients. Non-contrast enhanced chest CT scan was performed for the remaining four patients, on which typical changes of viral pneumonia, such as ground-glass opacity, were observed (Figure 3).

Oxygen therapy was administrated to three patients, including one in severe condition (PT 2) who required high-flow nasal cannula oxygen therapy (oxygen flow 40 L/min, oxygen concentration 40%), and the blood oxygen saturation of the patient was maintained at 99%. None of the 12 patients required ventilation support. Five patients only had mild disease, the course of which was self-limited. Glucocorticoid (40 mg, intravenous infusion twice a day) was only administrated to the patient with severe disease (PT 2).

## Discussion

The outbreak of the novel coronavirus SARS-CoV-2 has been declared by WHO as a public health emergency of international concern [1]. In view of its rapid spread, a better understanding of the epidemiology and clinical features of the disease is especially important, to aid early recognition and better containment. In the present study, most patients had a direct or indirect epidemiologic association with Wuhan, i.e. the patient had either returned from Wuhan or had been in contact with a confirmed patient who had returned from Wuhan. It is noteworthy that people are not necessarily infected by people who are obviously showing signs of the illness, but also by non-symptomatic carriers of the virus [8]. Thus, the pre-symptomatic transmission is a concern. Family aggregation is another epidemiologic feature observed in the present study. All these findings are consistent with other recent reports and indicate that COVID-19 is a highly contagious disease, the control of which requires early identification and strict quarantine [9–11].

Most of our patients developed only a mild disease with a self-limited disease course. Of the 12 patients observed in this study, only one elderly patient developed severe disease, which may be associated with existing underlying diseases and poor immunity. Consistent with the published findings, most patients had a normal WBC count and normal or reduced lymphocyte (LY) count [12]. It is worth mention that the patient with severe disease had a significantly low LY count, suggesting that lymphocyte reduction may correlate with disease severity.

SARS-CoV-2 infection was confirmed in all the patients via nucleic acid detection, but not all the patients showed a positive finding on radiography. Thus, chest x-ray may not be sensitive enough for identifying potential patients. On the other hand, all the patients underwent a chest CT scan and had a typical imaging finding of pneumonia. A recent study also reported that CT scan has a high sensitivity for the diagnosis of COVID-19 and can work as an auxiliary or even primary tool for COVID-19 detection when nucleic acid testing is not available [13].

SARS-CoV-2 is closely related to the SARS virus. They both belong to the genus human coronavirus (hCoV), which comprises both low- and high-pathogenic species. While a low-pathogenic coronavirus only infects the upper respiratory tract and causes mild, cold-like respiratory disease, highly pathogenic hCoVs such as SARS-CoV and Middle Eastern respiratory syndrome coronavirus (MERS-CoV) mainly infect the lower respiratory tract and can be fatal. Severe pneumonia induced by pathogenic hCoV is usually accompanied by rapid virus replication, massive inflammatory cell infiltration, and an elevated proinflammatory cytokine/chemokine response, leading to acute lung injury and respiratory distress syndrome. Therefore, during the rapid progression of the disease, glucocorticoid treatment can inhibit the overwhelmed inflammatory response and reduce damage to the lungs; this has been proved valid in the treatment of SARS [14].

Because of immune suppression, treatment with a steroid in MERS can result in delayed viral clearance and increased mortality and influenza infection [15]. A recent study reported that clinical evidence does not support corticosteroid treatment for lung injury due to SARS-CoV-2 [16]. Of the 12 patients in the present study, only one with severe disease was treated with steroids. In this patient, the pulmonary lesion was still not absorbed by the fifteenth day, and the pharynx remained positive for the nucleic acid throughout the course of the disease for 22 days. It seems that steroid treatments were not of significant benefit for this patient. Therefore, caution should be taken when considering corticosteroid administration in the treatment of COVID-19 pneumonia.

## Data Availability

The data that support the findings of this study are available from the corresponding author upon reasonable request.

## References

[1] Zhang H. Early lessons from the frontline of the 2019-nCoV outbreak. Lancet. 2020;395(10225):687. 32059798 10.1016/S0140-6736(20)30356-1 PMC7135082

[2] Wu F, Zhao S, Yu B, et al. A new coronavirus associated with human respiratory disease in China. Nature. 2020;579(7798):265–269. 32015508 10.1038/s41586-020-2008-3 PMC7094943

[3] Gorbalenya AE, Baker SC, Baric RS, et al. Severe acute respiratory syndrome-related coronavirus: the species and its viruses – a statement of the coronavirus study group. bioRxiv. 2020. DOI:10.1101/2020.02.07.937862

[4] World Health Organization. Director-general’s remarks at the media briefing on 2019-nCoV on 11 february 2020. [2020 Feb 11]. Available from: https://www.who.int/dg/speeches/detail/who-director-general-s-remarks-at-the-media-briefing-on-2019-ncov-on-11-february-2020

[5] Zhou P, Yang XL, Wang XG, et al. A pneumonia outbreak associated with a new coronavirus of probable bat origin. Nature. 2020;579(7798):270–273. 32015507 10.1038/s41586-020-2012-7 PMC7095418

[6] Lu CW, Liu XF, Jia ZF. 2019-nCoV transmission through the ocular surface must not be ignored. Lancet. 2020;395(10224):e39. 32035510 10.1016/S0140-6736(20)30313-5 PMC7133551

[7] Tang B, Bragazzi NL, Li Q, et al. An updated estimation of the risk of transmission of the novel coronavirus (2019-nCov). Infect Dis Model. 2020;5:248–255. 32099934 10.1016/j.idm.2020.02.001 PMC7029158

[8] Tong ZD, Tang A, Li KF, et al. Potential presymptomatic transmission of SARS-CoV-2, Zhejiang Province, China, 2020. Emerg Infect Dis. 2020;26(5). DOI:10.3201/eid2605.200198 PMC7181913 32091386

[9] Thompson RN. Novel coronavirus outbreak in Wuhan, China, 2020: intense surveillance is vital for preventing sustained transmission in new locations. J Clin Med. 2020;9:2. 10.3390/jcm9020498 PMC7073840 32054124

[10] Yu P, Zhu J, Zhang Z, et al. A familial cluster of infection associated with the 2019 novel coronavirus indicating potential person-to-person transmission during the incubation period. J Infect Dis. 2020. DOI:10.1093/infdis/jiaa077 PMC7107453 32067043

[11] Ki M, nCo VT.Epidemiologic characteristics of early cases with 2019 novel coronavirus (2019-nCoV) disease in Republic of Korea. Epidemiol Health. 2020;e2020007. DOI:10.4178/epih.e2020007 32035431 PMC7285424

[12] Song F, Shi N, Shan F, et al. Emerging coronavirus 2019-nCoV pneumonia. Radiology. 2020;295(1). 10.1148/radiol.2020200274 PMC7233366 32027573

[13] Ai T, Yang Z, Hou H, et al. Correlation of chest CT and RT-PCR testing in coronavirus disease 2019 (COVID-19) in China: A report of 1014 cases. Radiology. 2020:200642. 10.1148/radiol.2020200642 PMC7233399 32101510

[14] Stockman LJ, Bellamy R, Garner P. SARS: systematic review of treatment effects. PLoS Med. 2006;3(9):e343. 16968120 10.1371/journal.pmed.0030343 PMC1564166

[15] Arabi YM, Mandourah Y, Al-Hameed F, et al. Corticosteroid therapy for critically ill patients with Middle East respiratory syndrome. Am J Respir Crit Care Med. 2018;197(6):757–767. 29161116 10.1164/rccm.201706-1172OC

[16] Russell CD, Millar JE, Baillie JK. Clinical evidence does not support corticosteroid treatment for 2019-nCoV lung injury. Lancet. 2020;395(10223):473–475. 32043983 10.1016/S0140-6736(20)30317-2 PMC7134694

